# Ferric citrate hydrate is associated with a reduced cost of drugs and a smaller change in red blood cell distribution width

**DOI:** 10.1038/s41598-022-06261-0

**Published:** 2022-02-14

**Authors:** Kyoko Ito, Keitaro Yokoyama, Masaaki Nakayama, Masafumi Fukagawa, Hideki Hirakata

**Affiliations:** 1 Medical Affairs Department, Torii Pharmaceutical Co. Ltd, 3-4-1 Nihonbashi-Honcho, Chuo-ku, Tokyo, 103-8439 Japan; 2 Department of Health Science, The Graduate School, The Jikei University School of Medicine, Tokyo, Japan; 3 St. Luke’s International Hospital, St. Luke’s International University, Tokyo, Japan; 4 Division of Nephrology, Endocrinology and Metabolism, Tokai University School of Medicine, Isehara, Japan; 5 Fukuoka Renal Clinic, Fukuoka, Japan

**Keywords:** Health care, Nephrology

## Abstract

The ASTRIO study was a randomised, multicentre, 24-week study that compared the effects of ferric citrate hydrate (FC) and non-iron-based phosphate binders (control) on anaemia management in haemodialysis (HD) patients receiving erythropoiesis-stimulating agents (ESAs). In that study, FC reduced the doses of ESAs and intravenous iron without affecting haemoglobin (Hb); however, the cost-effectiveness of FC was unclear. We retrospectively implemented a cost-effectiveness analysis comparing the incremental cost-effectiveness ratios (ICERs) in FC (n = 42) and control (n = 40) groups in patients with serum phosphate and Hb controlled within the ranges of 3.5–6.0 mg/dL and 10–12 g/dL, respectively. Costs included drug costs of phosphate binders, ESAs, and intravenous iron. Elevated red cell distribution width (RDW) has been reported to be associated with mortality in HD patients and was therefore used as an effectiveness index. The mean (95% confidence interval) differences in drug costs and RDW between the FC and control groups were US$ − 421.36 (− 778.94 to − 63.78, *p* = 0.02) and − 0.83% (− 1.61 to – 0.05, *p* = 0.04), respectively. ICER indicated a decrease of US$ 507.66 per 1% decrease in RDW. FC was more cost-effective than non-iron-based phosphate binders. Iron absorbed via FC could promote erythropoiesis and contribute to renal anaemia treatment.

## Introduction

Chronic kidney disease (CKD) is associated with persistent renal damage and decreased renal function. According to the National Kidney Foundation, approximately 10% of the population worldwide suffers from CKD. CKD was the 17th leading cause of death in 1990 but became the 12th leading cause in 2017, according to a global burden of disease study^1^. Health care expenditure for patients with CKD in the USA exceeded US$ 79 billion in 2016, an increase of 23% from 2015, while the additional expenditure of US$ 35 billion for patients with end-stage renal disease (ESRD) increased the total expenditures related to both CKD and ESRD to over US$ 114 billion, representing 23% of total Medicare spending. Internationally, Taiwan, Japan, and the USA had the highest prevalence of ESRD in 2016, at 3,392, 2,599, and 2,199 patients per million people in the general population, respectively^2^. The Japanese Society for Dialysis Therapy (JSDT) survey reported that approximately 330,000 patients were receiving haemodialysis in Japan in 2016. According to the Organisation for Economic Co-operation and Development (OECD) Reviews of Public Health in 2019, haemodialysis costs in Japan were approximately 400,000 Japanese yen (approximately US$ 3,677) per patient per month, while the total medical costs for haemodialysis patients amounted to 1.57 trillion Japanese yen (approximately US$ 14.4 billion) per year^3^. Japan has a public financial support system for medical costs to reduce the financial burden on patients; however, national medical costs continue to increase, leading to financial concerns.

The progression of CKD is associated with mineral and bone disorders such as hyperphosphataemia and renal anaemia. Hyperphosphataemia causes ectopic calcification involving calcium deposition in various tissues other than bone, while renal anaemia reduces patient quality of life and worsens cardiac function. The Kidney Disease: Improving Global Outcomes (KDIGO) guidelines^4,5^ and JSDT guidelines^6–8^ recommend the use of phosphate binders to treat hyperphosphataemia and erythropoiesis-stimulating agents (ESAs) and iron preparations to treat renal anaemia. The International Dialysis Outcomes and Practice Patterns Study (DOPPS) for 2009–2015 reported that > 90% of haemodialysis patients in Japan, as well as in the USA and Europe, use ESAs^9^. In an attempt to maintain reasonable medical costs and curtail total medical costs for dialysis by 4%, the cost-reimbursement method for ESAs in dialysis patients in Japan was changed in 2006, from a fee-for-service payment to inclusion in the technical fee for dialysis^10^. In addition, the fee-for-service reimbursement for ESAs in the USA was replaced in 2011 with a fixed-sum bundled Medicare payment for dialysis service^11^.

Previous clinical studies in haemodialysis patients with hyperphosphataemia showed that iron-based phosphate binders, ferric citrate (Auryxia®, Akebia Therapeutics, Inc., MA, USA) and its hydrate (FC; Riona®, Torii Pharmaceutical Co., Ltd., Tokyo, Japan), not only improved serum phosphate levels but also increased haemoglobin (Hb) levels and reduced the required doses of ESAs and intravenous iron^12–14^. A previous study in the USA found that by reducing the need for ESAs and intravenous iron, ferric citrate reduced ESA costs by 8.15% and intravenous iron costs by 33.2% per 500 cases of ESRD, resulting in a savings of US$ 160.4 per patient per month compared with the costs associated with other phosphate binders (calcium acetate, sevelamer hydrochloride/carbonate, and lanthanum carbonate)^15^. A model predicted a cost reduction of US$ 1,585 per patient per year for ESAs and US$ 516 per patient per year for intravenous iron, resulting in a total savings of US$ 2,101 per patient per year^16^. One report noted that reducing the administration of ESAs and intravenous iron lowered the hospitalisation rate, which was expected to save an additional US$ 3,002 per patient per year in hospitalisation costs^17^.

The ASTRIO study was a randomised, multicentre, prospective, 24-week clinical study that compared the effects of FC and non-iron-based phosphate binders for hyperphosphataemia on the treatment of renal anaemia in Japanese patients receiving maintenance haemodialysis and ESA therapy. Although there were no changes in Hb or serum phosphate in either group, the doses of ESAs and intravenous iron were decreased in patients taking FC compared with those taking non-iron-based phosphate binders^18^. However, no cost-effectiveness analysis to date has compared the use of FC and non-iron-based phosphate binders in Japan in terms of drug costs and health outcomes. In the present study, we carried out a retrospective analysis of the ASTRIO study data to compare the cost-effectiveness of FC and non-iron-based phosphate binders in Japanese patients receiving maintenance haemodialysis.

## Results

### Subject characteristics

**Figure 1:**
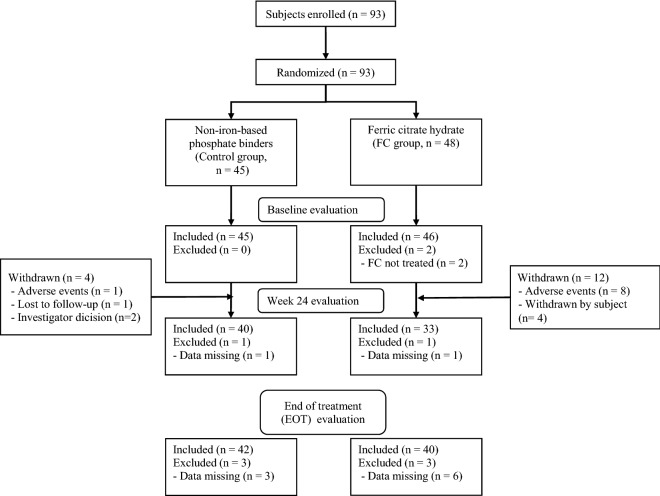
Patient flow in the ASTRIO study. End of treatment (EOT) was evaluated at week 24 or early discontinuation, if applicable.

Ninety-three haemodialysis patients with hyperphosphataemia were assigned in a 1:1 ratio to the FC group (n = 48) or control group (n = 45). Two subjects who dropped out before taking FC were excluded, and the remaining 91 subjects were included in the full analysis set (FAS). A further 12 subjects in the FC group and 4 in the control group dropped out during the study period, and 75 subjects (82%) therefore completed the 24-week administration period (Fig. 1). There were no significant differences in any subject characteristics, including age, main underlying disease, values of CKD-mineral bone disease (MBD) parameters, iron-related parameters, red blood cell parameters including red cell distribution width (RDW), and doses of ESAs, between the groups at baseline (Table 1).

**Table 1:** Baseline characteristics.

Characteristics	Control (n = 45)	FC (n = 46)	p value^1^
Age, yr	62.7 ± 12.7	63.3 ± 10.0	0.78
Body weight (before dialysis) (kg)	62.9 ± 13.6	60.0 ± 10.7	0.26
Sex (Male), n (%)	36 (80.0%)	30 (65.2%)	0.16^2^
IV iron preparations, Yes, n (%)	6 (13.3)	4 (8.7)	0.52
**Phosphate binder n (%)**^**3**^			
Calcium carbonate	30 (65.2)	28 (62.2)	0.83
Sevelamer hydrochloride	6 (13.0)	10 (22.2)	0.29
Bixalomer	4 (8.7)	5 (11.1)	0.74
Lanthanum carbonate hydrate	21 (45.7)	21 (46.7)	1.00
**ESA, n (%)**			
Epoetin alpha	21 (45.7)	14 (31.1)	0.20
Epoetin beta	1 (2.2)	3 (6.7)	0.36
Darbepoetin alpha	17 (37.0)	21 (46.7)	0.40
Epoetin beta pegol	7 (15.2)	7 (15.6)	1.00
Serum P (mg/dL)	5.2 (1.3)	5.4 (1.2)	0.42
Hb (g/dL)	10.5 (0.9)	10.5 (0.7)	0.78
TSAT (%)	21.2 (9.3)	23.0 (9.8)	0.36
Serum ferritin (ng/mL)	85.6 (85.8)	105.7 (85.5)	0.27
ESAs dose^4^ (IU/week)	5848.1 (4082.8)	5735.4 (4933.3)	0.91
MCV (fL)	93.8 (7.0)	94.1 (5.3)	0.83
MCH (fL)	30.6 (2.5)	30.8 (2.2)	0.63
RDW (%)	15.4 (1.8)	14.9 (1.6)	0.14

Values given as the mean ± standard deviation unless otherwise specified.

1) Student’s t test, 2) Fisher’s exact test, 3) Combination therapy were allowed.

4) Epoetin 200 IU = darbepoetin 1 μg = epoetin beta pegol 1 μg.

*FC* ferric citrate hydrate, *SD* standard deviation, *ESA* erythropoiesis-stimulating agent, *P* phosphate, *Hb* haemoglobin, *TSAT* transferrin saturation, *MCV* mean corpuscular volume, *MCH* mean corpuscular haemoglobin, *RDW* red blood cell distribution width.

### Changes in phosphate, Hb, iron-related parameters, and red blood cell-related parameters

There was no significant difference in the mean change in serum phosphate or Hb levels from baseline to the end of treatment (EOT) between the two groups. However, serum ferritin and transferrin saturation (TSAT) increased in the FC group, and the mean changes from baseline to EOT were significantly larger in the FC group than in the control group (+ 79.5 ng/mL, *p* < 0.01; + 9.0%, *p* < 0.01, respectively). Mean corpuscular volume (MCV) and mean corpuscular haemoglobin concentration (MCH) also increased in the FC group, with significantly larger mean changes from baseline to EOT in the FC group than in the control group (+ 3.9 fL, *p* < 0.01; + 1.51 pg, *p* < 0.01, respectively). The mean change in RDW from baseline to EOT was significantly lower in the FC group than in the control group (− 0.83%, *p* = 0.04)^18^.

### Drug cost calculation (phosphate binders, ESAs, and intravenous iron)

In line with the change from non-iron-based phosphate binders to FC at randomisation, the cost of phosphate binders increased in the FC group, while the cost of ESAs was reduced. The cumulative mean (95% confidence interval (CI)) difference in drug costs (phosphate binders, ESAs, and intravenous iron) using the mean drug prices for calcium carbonate and Epoetin alpha from baseline to EOT in the FC group compared with the control group was US$ − 421.36 (− 778.94 to − 63.78, *p* = 0.02) (Tables 2 and 3).

**Table 2:** The unit prices of drugs (US$).

	Unit	Brand drug price		Generic drug price		Mean price	Minimum price	Maximum price
**Phosphate binder**								
Calcium carbonate	500 mg tablet	0.06		0.05		0.06	0.05	0.06
	1 g fine granles	0.08		Not applicable (NA)		–	–	–
Sevelamer hydrochloride	250 mg tablet	0.28		NA		–	–	–
Bixalomer	250 mg capsule	0.28		NA		–	–	–
Lanthanum carbonate hydrate	250 mg tablet	1.56		NA		–	–	–
	500 mg tablet	2.29		NA		–	–	–
Ferric Citrate Hydrate	250 mg tablet	0.78		NA		–	–	–
**ESA**								
Epoetin alpha	750 IU	5.87	7.32	7.45	4.49	6.28	4.49	7.45
	1500 IU	9.55	15.86	13.92	7.18	11.63	7.18	15.86
	3000 IU	16.55	27.38	25.38	12.60	20.48	12.60	27.38
Epoetin beta	1500 IU	15.26		NA		–	–	–
	3000 IU	26.21		NA		–	–	–
Darbepoetin alpha	5 µg	12.35		NA		–	–	–
	20 µg	40.65		NA		–	–	–
	60 µg	103.68		NA		–	–	–
	120 µg	183.92		NA		–	–	–
Epoetin beta pegol	25 µg	61.25		NA		–	–	–
	50 µg	109.22		NA		–	–	–
	100 µg	194.32		NA		–	–	–
	200 µg	345.87		NA		–	–	–
**IV iron preparation**								
Saccharted ferric oxide	40 mg	0.56		NA		–	–	–

Source: Hokenyaku Jiten Aug 2016.

**Table 3:** Cumulative drug cost from baseline to the end of treatment (EOT).

Mean^1)^	Drug cost/4 weeks at Baseline (US$)				Cumulative drug cost from Baseline to EOT (US$)			
	Control (N = 42)	FC (N = 40)	Difference (FC-control)	p value^2)^	Control (N = 42)	FC (N = 40)	Difference (FC-control)	p value^2)^
	Mean (SD)		(95% CI)		Mean (SD)		(95% CI)	
Phosphate binder	92.69 (83.85)	85.17 (91.22)	− 7.52 (− 46.00, 30.96)	0.70	567.50 (469.86)	725.16 (408.39)	157.66 (− 36.21, 351.54)	0.11
ESA	223.60 (150.30)	208.28 (177.08)	− 15.32 (− 87.38, 56.74)	0.67	1392.67 (924.91)	816.99 (496.20)	− 575.68 (− 904.24, − 247.12)	< 0.01
Intravenous iron	0.08 (0.20)	0.06 (0.17)	− 0.02 (− 0.11, 0.06)	0.56	4.61 (5.20)	1.26 (2.49)	− 3.34 (− 5.15, − 1.54)	< 0.01
Total	316.37 (141.86)	293.51 (194.05)	− 22.86 (− 97.31, 51.58)	0.54	1964.77 (910.73)	1543.41 (696.33)	− 421.36 (− 778.94, − 63.78)	0.02

1) Mean drug prices were used to calculate the drug cost for Calcium carbonate and Epoetin alpha.

2) Student's t test.

In a sensitivity analysis, the drug cost using the minimum and maximum drug prices for calcium carbonate and Epoetin alpha were calculated. The cumulative mean (95% CI) differences in drug costs (phosphate binders, ESAs, and intravenous iron) from baseline to EOT in the FC group compared with the control group were US$ − 457.25 (− 807.84 to − 106.66, *p* = 0.01) and US$ − 388.25 (− 763.35 to − 13.14, *p* = 0.04), respectively.

### Achievement of RDW < 15.5%

There was no significant difference in the percentage of patients with RDW < 15.5% between the groups at baseline. However, the ratio became significantly higher in the FC group than in the control group after 12 weeks (72.7% in FC and 40.0% in control at 24 weeks, *p* < 0.01) (Table 4).

**Table 4:** Achievement ratio of RDW < 15.5%

Visit	Class	Control		FC		p value^1)^
		N	(%)	N	(%)	
Baseline	RDW < 15.5%	27	(60.0)	33	(71.7)	0.27
	RDW > = 15.5%	18	(40.0)	13	(28.3)	
Week 4	RDW < 15.5%	25	(55.6)	25	(64.1)	0.51
	RDW > = 15.5%	20	(44.4)	14	(35.9)	
Week 8	RDW < 15.5%	22	(53.7)	21	(60.0)	0.65
	RDW > = 15.5%	19	(46.3)	14	(40.0)	
Week 12	RDW < 15.5%	20	(48.8)	26	(76.5)	0.02
	RDW > = 15.5%	21	(51.2)	8	(23.5)	
Week 16	RDW < 15.5%	17	(41.5)	28	(84.8)	< 0.01
	RDW > = 15.5%	24	(58.5)	5	(15.2)	
Week 20	RDW < 15.5%	16	(41.0)	25	(78.1)	< 0.01
	RDW > = 15.5%	23	(59.0)	7	(21.9)	
Week 24	RDW < 15.5%	16	(40.0)	24	(72.7)	< 0.01
	RDW > = 15.5%	24	(60.0)	9	(27.3)	

1) Fisher's exact test.

### Cost-effectiveness analysis

During the ASTRIO study’s time frame (24 weeks), patients in the FC group achieved a 0.83% decrease in RDW, resulting in a savings of US$ 421.36 compared with the control group. The ICER resulted in a savings of US$ 507.66 per 1% decrease in RDW (Table 5).

**Table 5:** Incremental cost effectiveness ratio (ICER).

Change from baseline to EOT	Control	FC	Difference (FC-Control)	p value
	Mean (SD)		Mean (95% CI)	
Drug cost (US$)	1964.77 (910.73)	1543.41 (696.33)	− 421.36 (− 778.94, − 63.78)	0.02^1)^
RDW (%)	0.83 (2.11)	0.19 (1.59)	− 0.83 (− 1.61, − 0.05)	0.04^2)^
ICER	US$—507.66/-1% of RDW			

(Mean) 1) Student’s *t* test, 2) ANCOVA (covariate: baseline).

## Discussion

The current cost-effectiveness analysis using the ASTRIO study results for Japanese haemodialysis patients with hyperphosphataemia showed that FC was more cost-effective than continued use of non-iron-based phosphate binders (monotherapy or combination therapy of sevelamer hydrochloride, lanthanum carbonate hydrate, bixalomer, and precipitated calcium carbonate following in their package inserts) in terms of lower total drug costs (phosphate binders, ESAs, and intravenous iron) and a smaller change in RDW, as an effectiveness index correlated with all-cause mortality in haemodialysis patients. This was considered to be mainly caused by the promotion of erythropoiesis resulting from iron absorption during FC administration. This led to effective utilisation of ESA and produced mature red blood cells rich in haemoglobin.

Compared with the use of other phosphate binders, the use of ferric citrate reduced the costs of ESAs and intravenous iron in the USA^15,16^, while the present study showed a similar effect of FC on the cost of ESAs in Japan but a different effect on the cost of intravenous iron. This may be because intravenous iron is cheaper in Japan than in the USA, given that it is generally less widely used in Japan than in other countries. In haemodialysis patients, the reported mean doses of intravenous iron preparations (± standard deviation (SD)) were 33 ± 21 mg/week in Japan, 74 ± 47 mg/week in the EU, and 75 ± 52 mg/week in the USA. The reported rates of intravenous iron preparation use were 33% in Japan, 79% in the EU, and 77% in the USA, and the reported mean (SD) serum ferritin was 145 ± 205 ng/mL in Japan, 486 ± 380 ng/mL in the EU, and 774 ± 467 ng/mL in the USA^9^. The lower serum ferritin level in Japan than in other countries suggests that more haemodialysis patients in Japan are iron deficient, which could be an important reason that a smaller increase in serum ferritin resulting from iron absorbed during FC administration could promote erythropoiesis and produce mature red blood cells. The cost of phosphate binders was higher in the FC group than in the control group. In Japan, the rate of use of precipitated calcium carbonate, which is relatively cheap, was still high, which might have accounted for the higher cost of phosphate binders in the FC group.

We used RDW as an effective index in the ASTRIO study, and an increase in RDW was closely linked with all-cause mortality according to a systematic review and meta-analysis in CKD and haemodialysis patients^19^. The study also found no significant heterogeneity among the studies (CKD: *I*^2^ = 77%, *p* = 0.094, haemodialysis: *I*^2^ = 0.0%, *p* = 0.639)^19^. Although quality-adjusted life-years (QALYs) are frequently used as an effectiveness index, they have some disadvantages, including the fact that they may not accurately reflect the burden in short-term assessments^20^. Given that the ASTRIO study was a short-term (24-week) study, it was difficult to calculate QALYs reliably. A progressive increase in RDW, but not the baseline RDW, independently predicted mortality and cardiovascular events in patients with ESRD^21^. A higher RDW (≥ 15.5%) was also associated with a higher risk of mortality in haemodialysis patients^22^. In the present study, significantly more patients in the FC group achieved an RDW < 15.5% at 24 weeks compared with patients in the control group. The continued increase in RDW was therefore suppressed in the FC group compared with that in the control group, which was possibly associated with the reduced risks of mortality and cardiovascular events. The above results indicated that RDW was an appropriate effective index for use in the present study.

The change in MCV from baseline to week 24 increased and that in RDW decreased in the FC compared with that in the control group. This may have been a result of iron absorption resulting from the administration of FC. A negative correlation between MCV and cardiovascular events has been reported in CKD patients^23^, while the combination of a smaller change in RDW and a larger change in MCV improved the prediction of mortality risk^24^. The decreased change in RDW and increased change in MCV in the FC group compared with the control group in the present study suggested that the mortality risk might have been reduced. No previous study has reported on the effects of FC or ferric citrate on cardiovascular events and mortality risk; however, a randomised clinical study in the USA compared the administration of ferric citrate with usual care (phosphate binders other than ferric citrate, ESA, red blood cell transfusion, active vitamin D, and oral or intravenous iron permitted at the discretion of the treating nephrologist) for 9 months in predialysis CKD patients with an estimated glomerular filtration rate ≤ 20 mL/min/1.73 m^2^ and showed that significantly fewer patients in the ferric citrate group initiated dialysis and were hospitalised compared with the usual-care group^25^.

The present cost-effectiveness analysis based on the ICER calculated from the ASTRIO study results showed that FC was more cost-effective than non-iron-based phosphate binders in terms of overall drug costs and changes in RDW, which is associated with mortality in haemodialysis patients^19,26^. Interpreting this result in terms of both cost reduction and better health outcomes^27^ indicated that FC was economically advantageous.

This study had some limitations. This was a retrospective cost-effectiveness analysis using the results from the ASTRIO study, which had a limited sample size and limited study period^18^. Larger studies with longer evaluation periods are therefore needed to verify these results. We used RDW as an effectiveness index for ICER; however, further cost-effectiveness analyses using cardiovascular events or QALYs need to be performed in the future.

In conclusion, the current cost-effectiveness analysis of FC in Japanese haemodialysis patients with hyperphosphataemia showed that FC was more cost-effective than non-iron-based phosphate binders, both in terms of lower total drug costs (phosphate binders, ESAs, and intravenous iron) and in maintaining a lower RDW, suggesting that iron absorption resulting from FC administration promoted erythropoiesis and contributed to the treatment of renal anaemia.

## Material and methods

### Study design and scope

Using data from the ASTRIO study, we investigated the cost-effectiveness of FC compared with that of non-iron-based phosphate binders using the cost-effective analysis method (Table 6)^20,28^ to provide useful information for clinicians, patients, health care administrators, and public health officials.

**Table 6:** Study design and scope of the cost-effectiveness analysis.

Element	Content
Objective	To implement a cost-effective analysis for ferric citrate hydrate, an iron-based phosphate binder, compared with non-iron based phosphate binders, in patients with hyperphosphatemia and renal anaemia who were undergoing haemodialysis and ESA therapy
Interventions	Treatment for hyperphosphatemia and renal anaemia. According to the guidelines, for hyperphospatemia, phosphate binders (ferric citrate hydrate which iron based phosphate binder, or non-iron-based phosphate binders) have been used, and for renal anaemia, ESAs and intravenous iron have been used
Target population	Japanese patients with hyperphosphataemia and renal anaemia who were undergoing haemodialysis and ESA therapy
Perspective	Health care perspective
Time horizon	24 weeks
Costing year	2016
Currency	108.78 Japanese yen (JPY)/US$, which is yearly average TTM (telegraphic transfer middle rate) for 2016, quoted by MUFG Bank, Ltd., (Tokyo, Japan)
Study design	Trial-based cost-effective analysis
Data sources	Primary data of the ASTRIO Study, a randomised, prospective, multicentre, open-label, parallel-group, comparative study that investigated the influences of 24-week administration of an iron based phosphate binder of ferric citrate hydrate, or non-iron based phosphate binders on the treatment of renal anaemia in patients receiving maintenance haemodialysis and ESA
Cost measures	Drug costs of phosphate binders, ESA and intravenous iron
Outcome measures	Red cell distribution width (RDW), of which increases are associated with mortality in haemodialysis patients

### ASTRIO study

The ASTRIO study was a randomised, prospective, multicentre, open-label, parallel-group, comparative, 24-week study that investigated the effects of FC on the treatment of patients with renal anaemia receiving maintenance haemodialysis. Included patients were ≥ 20 years old, receiving ESA therapy, and taking non-iron-based phosphate binders (monotherapy or combination therapy of sevelamer hydrochloride, lanthanum carbonate hydrate, bixalomer, and precipitated calcium carbonate following the guidelines in their package inserts). Patients were randomly assigned to receive either FC (n = 48) or non-iron-based phosphate binders (control) (n = 45). According to the package insert, the starting dose of FC was 500 mg (2 tablets of 250 mg of FC containing approximately 60 mg ferric iron per tablet), taken three times daily (1500 mg/day) immediately after a meal. Following the latest JSDT guidelines^6,8^, serum phosphate levels and Hb levels were controlled between 3.5 and 6.0 mg/dL and 10 and 12 g/dL, respectively, during the study period. The primary endpoint was the change in ESA dose. The results of the ASTRIO study showed that compared with the control, FC significantly reduced the dose of ESAs from baseline to EOT (on the day of observation at week 24 or at discontinuation). The mean (± SD) changes in ESA doses were − 1,211.8 (± 3,609.5) IU/week in the FC group and + 1,191.55 (± 6,662.8) IU/week in the control group (*p* = 0.03). In addition, the mean (± SD) cumulative dose of intravenous iron from baseline to week 24 was significantly lower in the FC group (105.5 (± 199.5) mg) than in the control group (330.0 (± 355.7) mg) (*p* = 0.01).

The ASTRIO study was conducted between November 2015 and January 2017. The protocol was carried out in accordance with the Declaration of Helsinki and the Ethical Guidelines for Medical and Health Research Involving Human Subjects (December 22, 2014, Ministry of Health, Labour and Welfare) and was approved by the institutional review board of the Jikei University School of Medicine after obtaining written informed consent from all subjects (UMIN000019176, registered on October 1, 2015)^18^.

### Drug costs (phosphate binders, ESAs and intravenous iron)

Drug costs were calculated based on the actual administered drug costs (phosphate binders, ESAs, and intravenous iron) in the ASTRIO study. The drug prices were calculated according to the national health insurance drug price list in Japan in 2016 when the study was conducted (Table 2) and the amounts of phosphate binders (FC, sevelamer hydrochloride, lanthanum carbonate hydrate, bixalomer, precipitated calcium carbonate), ESAs (epoetin alpha, epoetin beta, darbepoetin alpha, epoetin beta pegol), and intravenous iron (saccharated ferric oxide, the only approved intravenous iron in Japan) administered during the study period.

Other medical costs, such as the costs paid by third-party payers (including insurance companies) and costs paid out-of-pocket by patients, were not affected in this study. In addition, no treatment-related serious adverse events occurred.

### Identification of effectiveness index

According to the recommendations for conduct, methodological practices, and reporting of cost-effectiveness analysis^20,28^, we conducted a literature search for systematic reviews and meta-analyses to determine if any of the following biomarkers evaluated in the ASTRIO study correlated with health outcomes related to all-cause mortality and/or cardiovascular events in haemodialysis patients: CKD-MBD (serum calcium and intact parathyroid hormone), iron-related parameters (serum ferritin and TSAT), and red blood cell parameters (MCV, MCH, and RDW). Because serum phosphate was maintained between 3.5 and 6.0 mg/dL and Hb was maintained between 10 and 12 g/dL in both groups, these parameters were excluded from our search. A systematic review and meta-analysis of nine studies including 117,047 CKD patients reported on the association between RDW and all-cause mortality in CKD patients. The results showed that for every 1% increase in RDW, the risk of all-cause mortality in haemodialysis patients increased by 36% (hazard ratio (HR) 1.36, 95% CI 1.20–1.53)^19^. Another report suggested that RDW was correlated with all-cause mortality and cardiovascular mortality in Japanese haemodialysis patients, with a 1% increase in RDW associated with a 21% increase in all-cause mortality (HR 1.21, 95% CI 1.03–1.43, *p* = 0.02) and a 27% increase in cardiovascular mortality (HR 1.27, 95% CI 1.04–1.56, *p* = 0.02)^26^. We could not find any systematic reviews or meta-analyses related to all-cause mortality and/or cardiovascular events in CKD and/or haemodialysis patients for other biomarkers. We therefore selected RDW and used changes in RDW from baseline to EOT in both groups as an effectiveness index. A higher RDW (≥ 15.5%) was also associated with a higher risk of mortality in haemodialysis patients^22^. To verify the appropriateness of RDW as an effectiveness index, we investigated the ratio of patients with an RDW < 15.5% and compared the ratios between the FC and control groups.

### ICER

The ICER is the ratio of the difference in costs between the two alternatives to the difference in effectiveness between the same two alternatives, as recommended by the Consolidated Health Economic Evaluation Reporting Standards (CHEERS) statement^28^. In the present study, the mean changes in drug costs and RDW from baseline to EOT were used to calculate the ICER:

$${\text{ICER}} = \frac{{{\text{Mean change in drug costs in FC group }}{-}{\text{ Mean change in drug costs in control group}}}}{{{\text{Mean change in RDW in the FC group }}{-}{\text{ Mean change in RDW in the control group}}}}.$$

### Analytical methods

In previous 28-week and 52-week studies of FC in Japanese haemodialysis patients with hyperphosphatemia^12^, the mean (SD) change in the dose of ESAs from baseline to week 24 was—1045.6 (2579.0) IU/week. The intended sample size was estimated to be 90 patients in total to provide 83.3% power to detect the difference in mean changes in the dose of ESAs from baseline to the EOT between the FC and control groups with a type 1 error of 5% by the Wilcoxon rank sum test. Based on the full analysis set (FAS) data from the ASTRIO study (FC (n = 46), control (n = 45)), we analysed data for drug costs and RDW that were available at both baseline and EOT (FC (n = 40), control (n = 42)) for the cost-effectiveness analysis. Differences in changes in mean drug costs between the two groups were tested using Student’s *t* tests with a two-sided significance level of 0.05. Differences in changes in mean RDW between the two groups were tested by analysis of covariance with baseline values as covariates at a two-sided significance level of 0.05. We calculated the rate of achievement of an RDW < 15.5% using the FAS data (FC (n = 46), control (n = 45)) and analysed the differences between the groups using Fisher’s exact test at a two-sided significance level of 0.05. Statistical analyses were performed using SAS version 9.3 or version 9.4 (SAS Institute Inc., Cary, NC, USA).

## Data Availability

The datasets generated and/or analysed during the study are available from the corresponding author on reasonable request.
